# Screen-Printed, Pure Carbon-Black Thermocouple Fabrication and Seebeck Coefficients

**DOI:** 10.3390/s19020403

**Published:** 2019-01-19

**Authors:** Christina Offenzeller, Marcel Knoll, Bernhard Jakoby, Wolfgang Hilber

**Affiliations:** Institute for Microelectronics and Microsensors, Johannes Kepler University Linz, 4040 Linz, Austria; marcel.knoll@jku.at (M.K.); bernhard.jakoby@jku.at (B.J.); wolfgang.hilber@jku.at (W.H.)

**Keywords:** carbon black, thermocouple, screen-printed, Seebeck coefficient

## Abstract

Thermocouples classically consist of two metals or semiconductor components that are joined at one end, where temperature is measured. Carbon black is a low-cost semiconductor with a Seebeck coefficient that depends on the structure of the carbon particles. Different carbon black screen-printing inks generally exhibit different Seebeck coefficients, and two can therefore be combined to realize a thermocouple. In this work, we used a set of four different commercially available carbon-black screen-printing inks to print all-carbon-black thermocouples. The outputs of these thermocouples were characterized and their Seebeck coefficients determined. We found that the outputs of pure carbon-black thermocouples are reasonably stable, linear, and quantitatively comparable to those of commercially available R- or S-type thermocouples. It is thus possible to fabricate thermocouples by an easily scalable, cost-efficient process that combines two low-cost materials.

## 1. Introduction

Temperature can be measured based on a large variety of temperature-dependent effects [1,2] such as the thermal expansion of solids [3] and liquids [4], the resistance of conductors [5], the current–voltage characteristic of a diode [6], Brownian motion [7,8], change in electrical permittivity [9,10], and a number of optical effects (e.g., temperature-dependent variation in refractive index [11]).

The thermoelectric effect is often used to measure temperature, in most cases by means of thermocouples. Thermocouples classically consist of two metal components that are connected at a junction. If the temperature at the junction differs from that at the other connection terminal of the metal, a thermoelectric voltage is generated that is proportional to the temperature difference between junction and connection terminal [12]. This voltage is due to electrons diffusing from the hotter to the colder side of the conductor, thus creating a difference in electric potential, where the resulting field-induced current balances the diffusion current in equilibrium [13]. Additionally, phonons contribute to the diffusion of electrons, as they “drag” them along while travelling towards the cold end of the conductor [14]. This drag force is not unique to conductors; it was first discovered in semiconductors, which also exhibit thermoelectric properties. It is of greater significance in semiconductors since there are fewer diffusing free electrons [12]. The material property that determines the magnitude of the thermoelectric voltage is the Seebeck coefficient. The generated voltage is proportional to the temperature difference and to the difference in Seebeck coefficients between the two connected materials. The Seebeck coefficient of a material depends on its Fermi level [13,15] and transport properties, and is thus influenced by impurities, lattice defects, and the associated scattering cross-sections [12].

In principle, thermocouples can be fabricated using any combination of two conductive or semiconducting materials as long as they have different Seebeck coefficients. A notable exception are superconductors, which exhibit no thermoelectric effect below their critical temperature. This allows not only the relative Seebeck coefficients (between two materials, usually given relative to platinum) but also absolute Seebeck coefficients to be measured [12].

In this paper, we present a cost-efficient method for fabricating thermocouples using low-cost materials: Carbon-black ink is a semiconducting material that is screen-printable and readily available in large quantities [16,17,18]. It is a *p*-type semiconductor, which, in combination with another conductor or semiconductor, can potentially form a low-cost thermocouple. The Seebeck coefficient of carbon strongly depends on its nanostructure: Single-walled carbon nanotubes, for instance, have been reported to exhibit a Seebeck coefficient of 45 µV/K [19], while multi-walled carbon nanotubes have a Seebeck coefficient of 11–19 µV/K [20]. Furthermore, making carbon-black powder screen-printable requires the addition of specific amounts of polymer binder and solvent. The proportion of binder and solvent needs to be sufficiently high for a paste with rheological properties suitable for screen-printing to form. At the same time, the proportion of carbon-black powder in the paste must be above the percolation threshold (i.e., the level above which interparticle contact between the carbon-black particles in the paste occurs) for the printing paste to exhibit semiconducting properties. Below this level, the paste is an electrical insulator [21]. Polymers, by their nature, have a high density of impurities and more defects than a pure metal or semiconductor, which again influences the thermoelectric properties [22]. Thus, a thermocouple can be formed from two different carbon-black screen-printing inks, with the advantage of combining a low-cost, easily scalable fabrication process with low-cost, commercially available materials.

The goal of this paper is to use established, commercially available carbon-black screen-printing inks for the fabrication of thermocouples solely containing carbon black as a conductor, which yields the advantage of using a screen printing protocol that has to be adapted to one particular material system (i.e., carbon-black inks) only. To do so, four different carbon-black screen-printing inks from renowned manufacturers were chosen and combined to form six different thermocouples. These thermocouples were screen-printed onto glass and then characterized. Additionally, a thermocouple consisting of carbon-black ink and gold was printed and its output tested. From the set of measurement data obtained, we determined the Seebeck coefficients of the carbon-black inks, which can be used both for the low-cost, large-scale fabrication of thermocouples and to predict the thermovoltages that might arise when carbon black is employed as a conductive material in environments with varying temperatures.

## 2. Materials and Methods

Four commercially available carbon-black screen-printing inks were tested:

- Electrodag PR-406B by Henkel (hereafter referred to as “406”)
- EDAG 109 E&C by Loctite (hereafter referred to as “109”)
- SD 2842 HAL by Peters (hereafter referred to as “2842”)
- SD 2843 HAL by Peters (hereafter referred to as “2843”)

Each of these screen-printing inks consists of carbon-black particles and a polymer binder matrix with solvent, making the carbon powder screen-printable. Some characteristic properties of the inks, as specified by the suppliers, are shown in Table 1.

The detailed composition of each ink is not disclosed by the respective manufacturer. However, it is known that there are some particular properties that typically differ from ink to ink, for example the type of polymer binder, the carbon-black concentration, and the particle size and structure. It is possible to a certain extent to develop an idea regarding some of these quantities from the Scanning Electron Microscope (SEM) images provided at the end of this section. 

**Figure 1:**
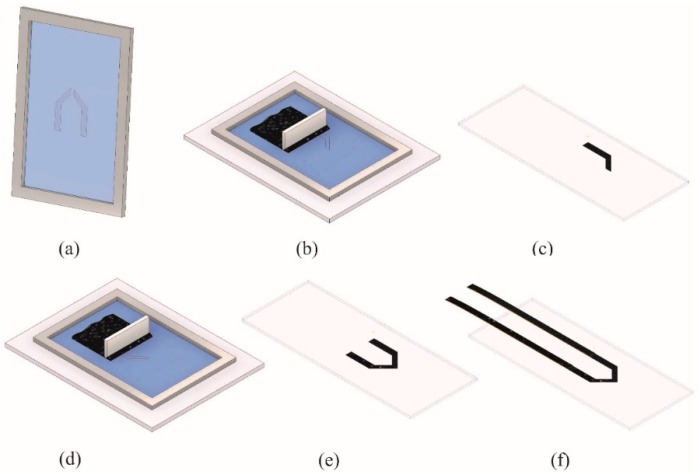
Fabrication process of a thermocouple. (**a**) Printing screen with the features to be printed; (**b**) printing of the first carbon-black ink; (**c**) the first carbon-black structure on glass substrate; (**d**) printing of the second carbon-black ink; (**e**) finished printed thermocouple; (**f**) thermocouple with carbon-black-coated Kapton strips as electrical connections.

The inks were then combined to form six thermocouples, each of which consisted of two different inks. To fabricate a thermocouple, we started by developing a photosensitive screen with 120 threads per cm PET mesh (Figure 1a). The first structure was printed onto a glass substrate using the first carbon-black ink (Figure 1b,c) and then dried at 150 °C for 5 min, after which the second structure was printed using the second carbon-black ink (Figure 1d). The two structures were printed such that they overlapped at the junction, that is, at the point at which temperature was to be measured (Figure 1e). Both inks were finally dried for 30 min at 150 °C. 

To be able to connect the thermocouples to the measurement setup, we fabricated electrical connections from Kapton® strips: Each printed carbon-black structure was connected with a Kapton strip coated with the respective carbon-black ink (Figure 1f). For this purpose, we used a doctor blade and then let the ink dry for 30 min at 150 °C.

Thus, we obtained six different thermocouples from four different inks. The additional thermocouple consisting of carbon-black ink (2842) and gold was fabricated as follows: The carbon-black feature was printed as described above, and the gold was applied as gold leaf with a fine brush. The connection for the gold structure was fabricated using a Kapton strip coated with gold leaf.

**Figure 2:**
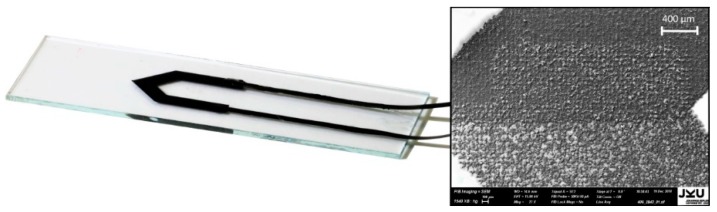
**Left**: Example thermocouple made from two different carbon-black inks on glass substrate with connection strips. **Right**: An SEM image of the area at which the printed features overlap.

An example of the resulting seven thermocouples can be seen in Figure 2 along with an SEM image of the overlapping printed features.

**Figure 3:**
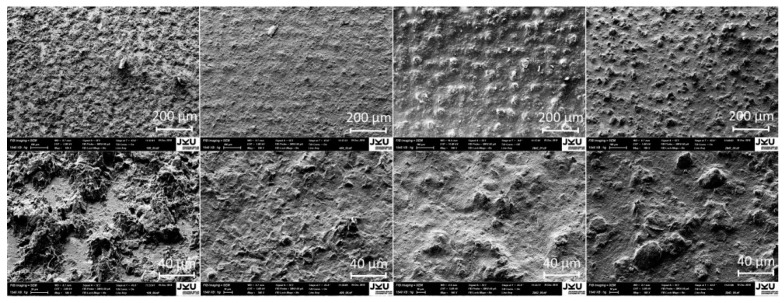
Scanning Electron Microscope (SEM) images of the printed inks. From left to right: inks 109, 406, 2842, and 2843. An image with a lower (**top**) and a higher (**bottom**) magnification was made of each ink.

Scanning electron microscopy was performed to qualitatively characterize the structural properties of the printed and dried inks. The resulting images are shown in Figure 3. It can be seen that the amount of polymer binder in ink 109 is apparently far lower than in inks 2842 and 2843, while ink 406 contains the highest fraction of binder. The carbon particles in inks 2842 and 2843 are agglomerated and significantly extend out of plane, whereas in ink 109 they appear to be very thin. Therefore it is obvious that the underlying structure and concentration of the carbon particles differ from ink to ink.

## 3. Results

This section first describes the measurement setup and then presents the measurement results of three pure carbon-black thermocouples and carbon-black and gold thermocouple. From these we determined Seebeck coefficients of the four carbon-black inks. To verify the results, we tested the remaining three possible combinations of carbon-black inks, measured their outputs, and compared them to the theoretical outputs predicted from the Seebeck coefficients.

### 3.1. Measurement Setup

**Figure 4:**
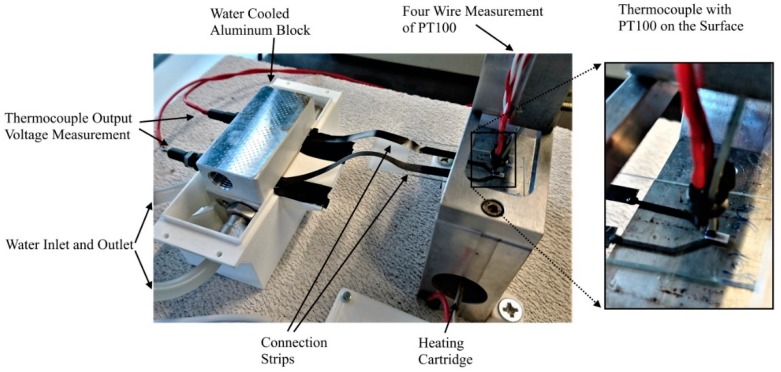
Measurement setup for characterizing the thermocouples. A thermocouple was placed on a metallic block that was heated from the inside by a heating cartridge. A PT100 thermistor was attached to the surface of the thermocouple junction and read out by four-wire measurement using a digital multimeter. The connections of the thermocouple were placed inside a water-cooled aluminum block and connected to a digital multimeter to measure the thermocouple output voltage.

Figure 4 shows the setup for testing the fabricated thermocouples: The glass substrate with the thermocouple was placed on an aluminum block containing a heating cartridge. The cartridge heated the block—and thus the thermocouple—from below. A PT100 thermistor placed on the surface of the thermocouple structure, directly atop the junction, was read out by four-wire measurement using a digital multimeter (DMM7510 by Keithley) to determine the temperature at the junction. The thermocouple connections (Kapton strips) were placed in a second aluminum block, through which water was pumped, maintained at 25 °C by a water bath to ensure that the aluminum block (and thus the connections) also remained at the same temperature. The connections were contacted and read out with a digital multimeter (DMM7510 by Keithley). 

### 3.2. Characterization of the Pure Carbon-Black Thermocouples and Determination of the Seebeck Coefficients

**Figure 5:**
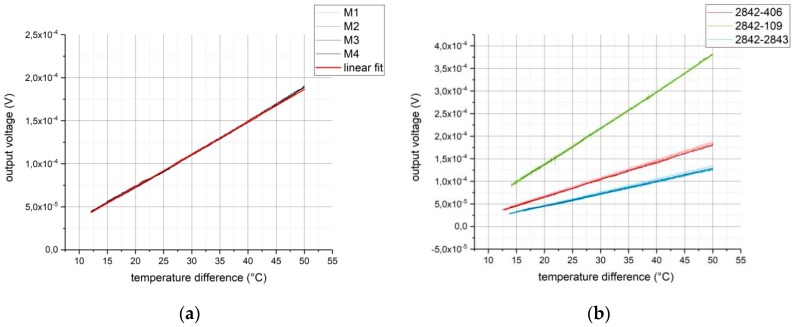
(**a**) Output voltage as a function of temperature difference between junction and connection. Four measurement cycles, M1-M4 (black), of the 2842-gold thermocouple and the corresponding linear fit (red); (**b**) output voltage as a function of temperature difference: outputs of thermocouples 2842-406 (red), 2842-406 (green), and 2842-2843 (blue). Each thermocouple was tested in four measurement cycles.

First, three thermocouples containing carbon black 2842 (i.e., 2842-406, 2842-109, and 2842-2843) and the 2842-gold thermocouple (i.e., 2842-Au) were characterized. To this end, we kept the connection temperature constant at 25 °C. The junction was heated to 75 °C (corresponding to a temperature difference of 50 °C). While this testing setup can be used up to a temperature of 400 °C, the inks were only characterized below their maximum operating temperature, which is on the order of 150 °C. The measured data were recorded during the cooling process. The measurement results are shown in Figure 5. 

As mentioned in the introduction, the output voltage $U_{1,2}$ of a thermocouple is proportional to the difference in the Seebeck coefficients of the two materials ($S_{1}$ and $S_{2}$) and the difference in temperature between junction and connection ∆T:

(1) $$U_{1,2}=\left(S_{2}-S_{1}\right)\cdot ∆T$$

The output voltage of the 2842-gold thermocouple is shown in Figure 5a. Four measurement cycles of heating and allowing the sample to cool down were performed.

The output voltage of the thermocouple was very stable over the four measurement cycles and largely linear. A linear fit (red function in Figure 5a) was calculated to determine the Seebeck coefficient of the first carbon black ink (i.e., 2842):

(2) $$U_{2842,Au}=\left(S_{Au}-S_{2842}\right)\cdot ∆T,$$

where $U_{2842,Au}$ is the output voltage of the 2842-gold thermocouple, $S_{2842}$ is the Seebeck coefficient of carbon black 2842, and $S_{Au}$ is the Seebeck coefficient of gold, which is known to be 1.94 µV/K (in absolute values, i.e., not relative to platinum) [23]. 

Dividing Equation (2) by the temperature difference gives the slope in Figure 5a, which equates to the difference in Seebeck coefficients. Therefore, the Seebeck coefficient of carbon black 2842 can be determined from the slope of the linear fit to be $-1.85\;\pm 1.86\cdot 10^{-3}$ µV/K.

Figure 5b presents the measurement data for the three pure carbon-black thermocouples featuring 2842. Each measurement cycle was repeated four times. It can be seen that the carbon-black inks have significantly different Seebeck coefficients. Calculating the slope of each linear fit allowed the difference in Seebeck coefficient between 2842 and the other carbon-black inks—and analogously also those of the remaining three carbon black inks—to be determined. The results are shown in Table 2.

The Seebeck coefficients of the carbon-black inks vary from −1.85 µV/K to +6.12 µV/K. As previously mentioned, there are several reasons for this effect: Nanostructure and size of the particles in the polymer matrix and the content of semiconducting material differ between the screen-printing inks. Furthermore, the polymer matrix affects the Seebeck coefficient via the level of impurity and the number of scattering centers in the ink.

In order to verify the results, three thermocouples of the possible combinations of inks 406, 109, and 2843 were characterized and then fabricated.

### 3.3. Verification of the Seebeck Coefficients

The outputs of thermocouples 406-109, 2843-109, and 2843-406 can be predicted theoretically from Equation (1):

(3) $$\frac{U_{406,109}}{∆T}=\left(S_{109}-S_{406}\right)=4.07\;µV/K$$

(4) $$U_{2843,109}/∆T=\left(S_{109}-S_{2843}\right)=5.19\;µV/K$$

(5) $$U_{2843,406}/∆T=\left(S_{406}-S_{2843}\right)=1.12\;µV/K$$

**Figure 6:**
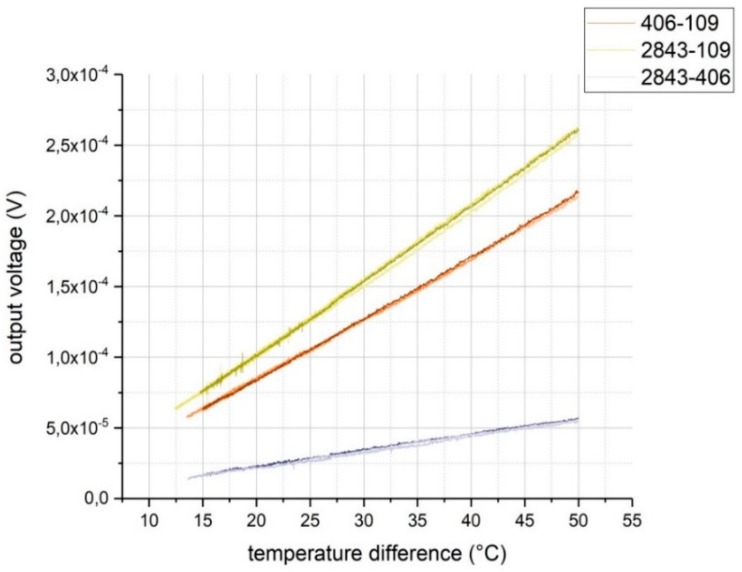
Output voltages of thermocouples 406-109, 2843-109, and 2843-406 as functions of temperature difference between junction and connections; four measurement cycles per thermocouple.

The results of measuring their outputs are plotted in Figure 6. 

Again, the output voltage as a function of temperature difference is fairly linear and the slopes differ between thermocouples.

For each of the thermocouples a linear fit was calculated. The slopes of the fits represent the output voltages per Kelvin, which we compared to the theoretical values in Table 3.

As can be seen, the values measured for the 2843-109 and 2843-406 thermocouples deviate by less than 1% from the predicted values. For the 406-109 thermocouple, the deviation amounts to 5.4%. Possible reasons for this lie in the origin of the varying Seebeck coefficients of carbon black: Since the concentration of carriers and defects in the material at the junction significantly influences the Seebeck coefficent, the deviation may be due to inhomogeneous carbon-particle distribution.

## 4. Discussion

In this paper, we have presented screen-printed thermocouples that consist exclusively of carbon-black inks. We have shown that by screen printing two carbon inks it is possible to produce a stable thermocouple that exhibits a linear output voltage which is comparable to that of classical (e.g., platinum/rhodium–platinum) thermocouples [25]. The Seebeck coefficients of four commercially available carbon-black screen-printing inks were determined. Based on this data, it is possible to produce low-cost thermocouples by an easily scalable process using readily available materials. Particular attention should be paid to temperature variations when using carbon-black structures as a conductive material (e.g., in sensor applications) connected, for instance, to copper wires. Unless accounted for, associated spurious thermovoltages may significantly distort measurement results.

## Figures and Tables

**Table 1:** Typical characteristic properties of the used inks.

Ink	Solids Content by Weight (%)	Viscosity (Pa∙s)	Density (g/cm^3^)	Sheet Resistivity (Ω/sq/25 µm)
406	56–60	35–45	1.25	<10
109	40	2.2	1.03	<30
2842	76–84	29	1.37	14
2843	73–77	17	1.27	13

**Table 2:** Materials used and their Seebeck coefficients and additional values of typical thermoelectric materials for comparison.

Material	Seebeck Coefficient (µV/K)
Gold	−1.94 [23]
2842	−1.85±0.002
406	2.05±0.002
109	6.12±0.003
2843	0.93±0.002
Platinum	−5.28 [24]
Palladium	−9.00 [24]
Copper	1.83 [24]

**Table 3:** Comparison of predicted and measured output voltages per Kelvin.

Thermocouple	Output (Predicted) (µV/K)	Output (Measured) (µV/K)	Deviation from the Predicted Value (%)
406-109	4.07	4.29	5.4
2843-109	5.19	5.24	0.96
2843-406	1.12	1.13	0.01
